# Feasibility of a virtual reality intervention targeting distress and anxiety symptoms in patients with primary brain tumors: Interim analysis of a phase 2 clinical trial

**DOI:** 10.1007/s11060-023-04271-0

**Published:** 2023-03-08

**Authors:** Amanda L. King, Kayla N. Roche, Heather E. Leeper, Elizabeth Vera, Tito Mendoza, Kelly Mentges, Alvina A. Acquaye-Mallory, Kendra A. Adegbesan, Lisa Boris, Eric Burton, Anna Choi, Ewa Grajkowska, Tricia Kunst, Jason Levine, Nicole Lollo, Hope Miller, Marissa Panzer, Marta Penas-Prado, Valentina Pillai, Lily Polskin, Jennifer Reyes, Solmaz Sahebjam, Macy L. Stockdill, Brett J. Theeler, Jing Wu, Mark R. Gilbert, Terri S. Armstrong

**Affiliations:** 1 Neuro-Oncology Branch, National Cancer Institute, National Institutes of Health, Bethesda, USA; 2 Frederick National Laboratory for Cancer Research, Leidos Biomedical Research, Inc., Frederick, USA; 3 Center for Cancer Research Office of Information Technology, National Cancer Institute, National Institutes of Health, Bethesda, USA; 4 Uniformed Services University of the Health Sciences, Bethesda, USA; 5 Office of Patient-Centered Outcomes Research, National Cancer Institute, National Institutes of Health, 9030 Old Georgetown Road, Bethesda, MD 20892 USA

**Keywords:** Virtual reality, Primary brain tumor, Distress, Anxiety

## Abstract

**Purpose:**

Cancer patients experience distress and anxiety when undergoing imaging studies to monitor disease status, yet these symptoms are not always appropriately identified or well-managed. This interim analysis of a phase 2 clinical trial explored feasibility and acceptability of a virtual reality relaxation (VR) intervention for primary brain tumor (PBT) patients at the time of clinical evaluation.

**Methods:**

English speaking, adult PBT patients with previous reports of distress and upcoming neuroimaging were recruited between March of 2021 and March 2022. A brief VR session was done within 2 weeks prior to neuroimaging with patient-reported outcomes (PROs) collected before and immediately post-intervention. Self-directed VR use over the next 1 month was encouraged with additional PROs assessments at 1 and 4 weeks. Feasibility metrics included enrollment, eligibility, attrition, and device-related adverse effects with satisfaction measured with qualitative phone interviews.

**Results:**

Fifty-five patients were approached via email, 40 (73%) responded and 20 (50%) enrolled (9 declines, 11 screen fails). 65% of participants were ≤ 50 years, 50% were male, 90% were White/non-Hispanic, 85% had good KPS (≥ 90), and most were on active treatment. All patients completed the VR intervention, PROs questionnaires, weekly check-ins, and qualitative interview. Most (90%) reported frequent VR use and high satisfaction and only 7 mild AEs were recorded (headache, dizziness, nausea, neck pain).

**Conclusion:**

This interim analysis supports feasibility and acceptability of a novel VR intervention to target psychological symptoms for PBT patients. Trial enrollment will continue to assess for intervention efficacy.

**Trial Registration:**

NCT04301089 registered on 3/9/2020.

**Supplementary Information:**

The online version contains supplementary material available at 10.1007/s11060-023-04271-0.

## Introduction

The National Comprehensive Cancer Network (NCCN) defines psychological distress in cancer patients as a multifactorial unpleasant emotional experience of a psychological, social, and/or spiritual nature which can affect a patient’s ability to effectively cope with their cancer diagnosis, its physical symptoms, treatment-related toxicities, and diagnostic imaging [1–3]. Past work has shown that patients with brain tumors have some of the highest prevalence of clinically significant distress among all solid tumor patients [4, 5], yet this symptom is not always appropriately identified or well-managed in clinical practice [6]. Distress exists along a continuum that ranges from normal adjustment to life stressors to more pervasive adjustment, anxiety and depressive disorders on the severe end of the spectrum [7]. While experiencing distress at some point during the cancer journey is inevitable, ideally the goal is to identify and treat distress in cancer patients early before it progresses to more severe psychological problems that are more difficult to treat and more likely to negatively impact clinical outcomes [8, 9].

After patients with primary brain tumors are diagnosed, they typically face an overall poor prognosis with a challenging clinical course and high symptom burden [10, 11]. In addition to the stress related to diagnosis, surgery, and treatment, these patients face an incurable tumor that requires lifelong surveillance imaging to monitor for likely recurrence. The term “scanxiety” describes the distress patients can experience related to significant anxiety surrounding the time that they have diagnostic imaging performed ahead of their clinical evaluations [12]. While there are some individuals who are anxious about being in the MRI scanner due to claustrophobia, others are more anxious about the results of the scan and the implications for their survival trajectory. Patients with primary brain tumors experience significant uncertainty surrounding their illness and it has been proposed that intervening upon distress and other mood-related symptoms (i.e. anxiety, depression) may modify the negative impact on subsequent symptom burden [13]. Therefore, targeting distress in brain tumor patients at the time of clinical evaluation may also improve both their psychological and physical health.

In clinical practice, the common approach is to refer highly distressed patients to mental health professionals, social workers, and/or chaplains for further evaluation so they can get targeted support to address their psychological needs. One major problem with this strategy is that despite well-intended referrals, less than half of patients elect to utilize these services when offered [4]. One innovative strategy that has been increasingly used in clinical populations is virtual reality (VR), which offers immersive computer-graphic or video-based environments that allow users to feel actually present in a virtual world [14–16]. VR has been implemented in a variety of adult and pediatric clinical populations, but has been utilized infrequently in oncology patients. A recent systematic review summarizing use of VR in solid tumor patients demonstrated promising improvements in distress, anxiety, and depression while also providing helpful distraction from unpleasant procedures or treatments [17], though brain tumor patients have been largely excluded from past trials and continue to be understudied for psychosocial interventions.

The primary aim of this phase 2 trial was to determine the feasibility and acceptability of a VR intervention to target distress and anxiety symptoms in a PBT patient population at the time of clinical evaluation. We hypothesized that (1) the VR intervention would be feasible in this population based on established parameters of eligibility, enrollment, device compliance and adverse events, and completion of PROs assessments, and (2) patients would report high satisfaction with the intervention. There are additional secondary and exploratory aims of this trial (Supplementary Fig. 1), which will be reported elsewhere following trial completion.

## Methods

### Study design

This was a phase 2 clinical trial with a single arm experimental design (Supplementary Fig. 2) which evaluated the feasibility of a VR intervention to improve distress and anxiety symptoms for PBT patients at the time of clinical evaluation. While initially intended to be conducted in-person, due to the COVID-19 pandemic all aspects of this trial were conducted remotely via telehealth with participants using VR in their home.

### Participants and recruitment

The study population was comprised of patients who were actively enrolled on the Neuro-Oncology Branch Natural History Study trial (NCT02851706) for primary central nervous system tumors [18]. Patients were screened for trial eligibility based on pre-defined criteria, which are outlined in Table 1. Potential patients were identified by screening for those scheduled for follow-up disease evaluation with subsequent review of their clinic notes in the electronic medical record and discussion with their clinicians. Patients were recruited during clinic or telehealth visits, as well as via email reach-outs using a study flyer. Interested patients who met eligibility criteria were consented remotely via telehealth.

**Table 1:** Eligibility criteria for VR trial

Inclusion criteria	Exclusion criteria
1. Diagnosed with PBT (brain + spine disease permissible)	1. Lack of definitive tissue diagnosis (no past surgery or biopsy)
2. Enrolled on NHS trial at NIH	2. Recent cranial surgery ≤ 2 weeks prior to VR intervention
3. Age ≥ 18 years old	3. Scalp wound healing issues that might interfere with VR headset use
4. Able to understand & sign informed consent	4. Pre-existing diagnosis of epilepsy (prior to brain tumor diagnosis) or recent seizures ≤ 6 weeks prior to VR intervention
5. Can reliably self-report symptoms (based on clinician assessment)	5. Diagnosis of GAD, PTSD, claustrophobia, or panic disorder
6. Upcoming clinic or telehealth appointment with associated MRI scan	6. Hypersensitivity to motion or currently experiencing severe nausea
7. Reported distress ≥ 1 on past symptom questionnaires	7. Visual deficits, including hemianopsia, diplopia, and agnosia, that may interfere with VR experience

*PBT* primary brain tumor; *NHS* Natural History Study; *NIH* National Institutes of Health; *MRI* magnetic resonance imaging; *VR* virtual reality; *GAD* generalized anxiety disorder; *PTSD* post-traumatic stress disorder

### VR intervention

Research staff demonstrated use of the VR headsets with the patients in a telehealth meeting prior to the initial intervention. Once all baseline assessments were collected, patients completed a brief, self-selected 5-min VR intervention under remote supervision by study staff. Staff remained in the telehealth meeting with the patients during the VR intervention so they could monitor for any technology issues or device-related AEs.

**Fig. 1:**
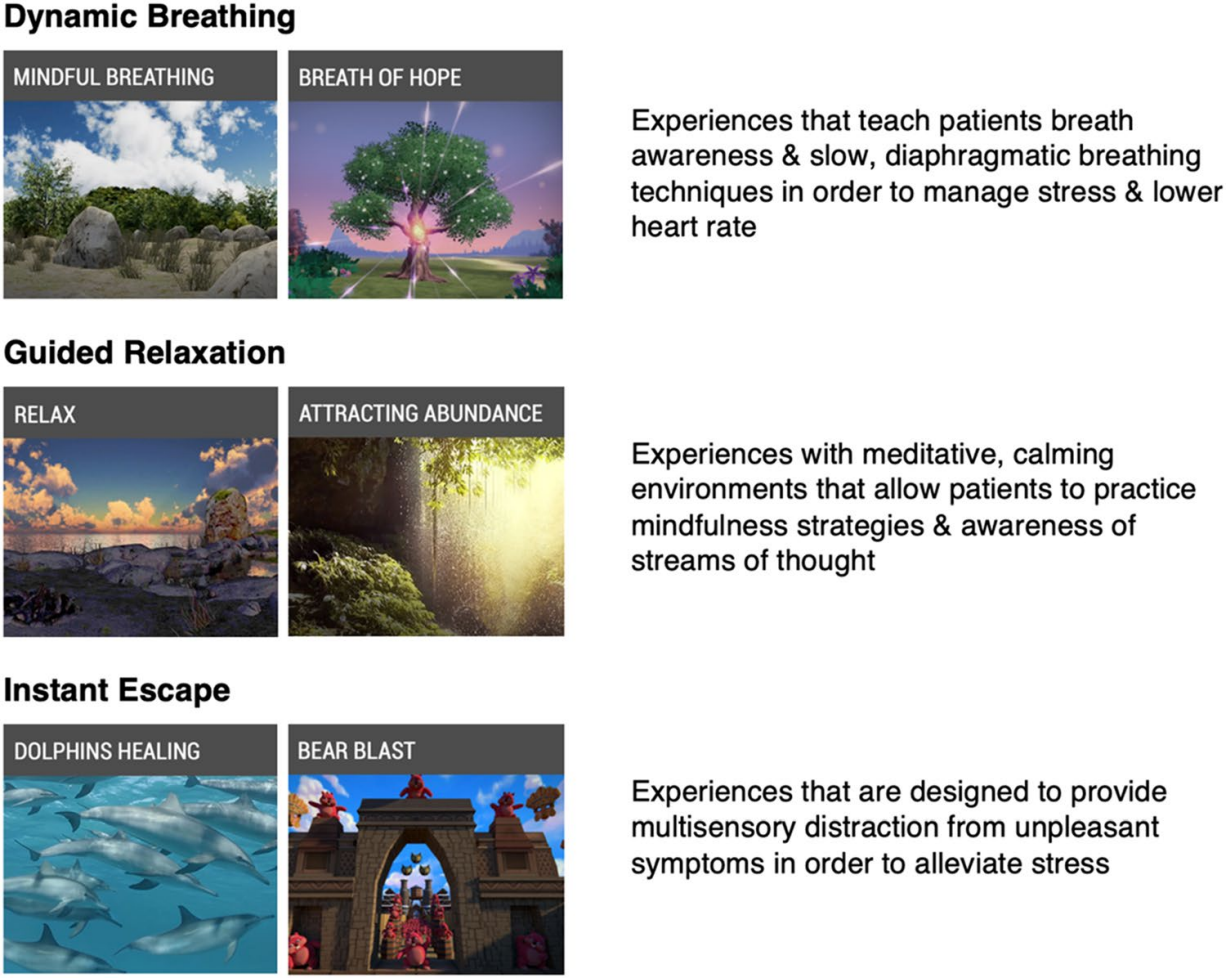
AppliedVR™ virtual scenarios on Pico G2 4K headset. The dynamic breathing scenarios, which make use of a breath shield attachment, guide the participant to take slow, deep breaths in order to slow the heart rate and induce relaxation as the environment seen changes based on the breathing pattern. Guided relaxation scenarios are meditative in nature and promote mindfulness and bringing attention to unhelpful thoughts and emotions that participants might be experiencing. Instant escape scenarios allow distraction through exploration of immersive environments, including ocean-based experiences, travel to various locations around the world, and interactive games

The VR headset used in this trial is the Pico G2 4K device, which is an immersive, lightweight, stand-alone headset that comes with an orientation-tracked controller and does not require a smartphone or a PC to function. This headset can be used via “gaze mode” or “controller mode” where the user can make selections on the screen by either directing their gaze at a particular item or by pointing at it using the remote controller. Additionally, there is a breath shield attachment on the front of the headset that can detect breathing patterns of the user and will change the virtual environment experienced if a breath-based scenario is chosen. The VR software loaded on the headset was designed by AppliedVR™ for use within clinical populations and aims to target unpleasant symptoms and promote relaxation. There are a total of 41 scenarios on the VR headset that fall within 3 main categories: (1) Dynamic Breathing, (2) Guided Relaxation, and (3) Instant Escape, shown in Fig. 1. While there are several interactive games on the VR device, participants were instructed not to choose these during the initial VR intervention since they tend to be more stimulating than anxiolytic.

Following the initial VR intervention, patients had self-directed VR use for the 1 month period they were on study and could choose any scenario available on the headset. Study staff conducted weekly check-ins to help troubleshoot any technological questions, to ask about device-related AEs, and to ask how often they used VR during the previous week. Other members of the household, including caregivers, were permitted to use the VR headset and patients were asked to inform us if this occurred, though no data was collected from those individuals.

### Measures

#### Feasibility and acceptability

This study will be considered successful if the following feasibility and acceptability metrics are met: 80% of approached eligible patients agree to participate in the trial, 70% compliance with VR headset use during the initial intervention, 70% of PROs are completed, no grade 3 or higher device-related adverse effects (AEs) reported, and high patient satisfaction with the intervention, which is determined by responses obtained during the qualitative interview and the Was It Worth It (WIWI) questions.

#### Patient-reported outcomes

Study outcome measures were collected using validated, patient-reported instruments. Device-related AEs were a primary outcome, distress and anxiety symptoms were secondary outcomes, and loneliness, financial toxicity, and adjustment disorder were exploratory outcomes. Table 2 outlines additional details about the PROs instruments used in this trial.

**Table 2:** PROs measures included in trial

Measures	Description	Clinical cut-offs
Primary		
PRO-CTCAEs [19]	5 items (related to VR use) with option to add items, 5-point Likert scale or 0/1 for absent/present	1 = mild 2 = moderate 3 = severe 4 = life-threatening 5 = death
Secondary		
NCCN Distress Thermometer [20]	1 item, 11-point Likert scale with accompanying Problem List	≥ 5 = moderate-severe
STAI-6 (*S*-scale) [21, 22]	6 items from *S*-scale, 4-item Likert scale (total score converted to range from 20 to 80)	≥ 40 = clinically significant
Exploratory		
UCLA Loneliness Scale [23, 24]	20 items, 4-point Likert scale	20 to 34 = low 35 to 49 = moderate 50 to 64 = moderately high 65 to 80 = high
COST Questionnaire [25]	11 items, 5-point Likert scale	not established
ADNM-20 [26]	Stressor & Item lists, 4-point Likert scale	≥ 47.5 = high risk

*PRO-CTCAEs* Patient-Reported Outcomes version of the Common Terminology Criteria for Adverse Events; *AE* adverse event; STAI-6: State-Trait Anxiety Inventory 6-item; *S*-scale: “state” anxiety subscale of STAI-6; *UCLA* University of California – Los Angeles; *COST* Comprehensive Score for Financial Toxicity; *ADNM-20* Adjustment Disorder New Module-20

#### Qualitative assessment

A 7-item semi-structured questionnaire was used during a phone interview with trial participants 1 week following the initial VR intervention in order to assess patient satisfaction with the intervention, feedback about the device, adverse effects related to device use, and the patient’s experience during the COVID-19 pandemic related to their psychological health. The interview concluded with 4 yes/no WIWI questions that further assessed satisfaction with the VR intervention. The phone interviews were recorded and the content transcribed to allow for qualitative thematic analysis. For the purposes of this interim analysis, responses to the WIWI questions were used to report patient satisfaction and the results from qualitative thematic analysis will be reported elsewhere.

#### Correlative biomarkers

This trial has optional collection of salivary stress biomarkers, including salivary cortisol, dehydroepiandrosterone-sulfate, and salivary alpha amylase, which are collected by patients at their home with kits supplied by the study team. Due to restrictions related to the COVID-19 pandemic, no saliva has been collected to date.

### Data management and monitoring

The PROs data from the questionnaires was collected via the Scribe electronic interface using links emailed to participants at the 4 study timepoints (baseline, immediate post-VR intervention, 1 week post-VR intervention, and 1 month post-VR intervention). All trial data was exported into a password-protected internal database and audited for errors by trained data analysts. To protect confidentiality, patient identifiers are stored in a separate location from the research data and only the key study personnel have access to identifying information.

Device-related AEs were assessed via the PRO-CTCAE questionnaires and through patient report during weekly check-ins with the study team. In the event that the participant reported any adverse effects, either during the intervention or with ongoing VR use at home, they were instructed to remove the VR headset and allow time to recover from the symptoms. If their symptoms persisted despite a break from using the device, their clinical team was notified, VR use was discontinued, and they completed follow-up PROs assessments, per investigator and clinician discretion.

### Statistical analysis

To evaluate the feasibility of the VR intervention, descriptive statistics were used to summarize rates of recruitment and retention, data completion, compliance, device-related AEs, and patient satisfaction. Analysis of secondary and exploratory aims will be reported elsewhere following recruitment of the full cohort.

## Results

### Feasibility of recruitment

**Fig. 2:**
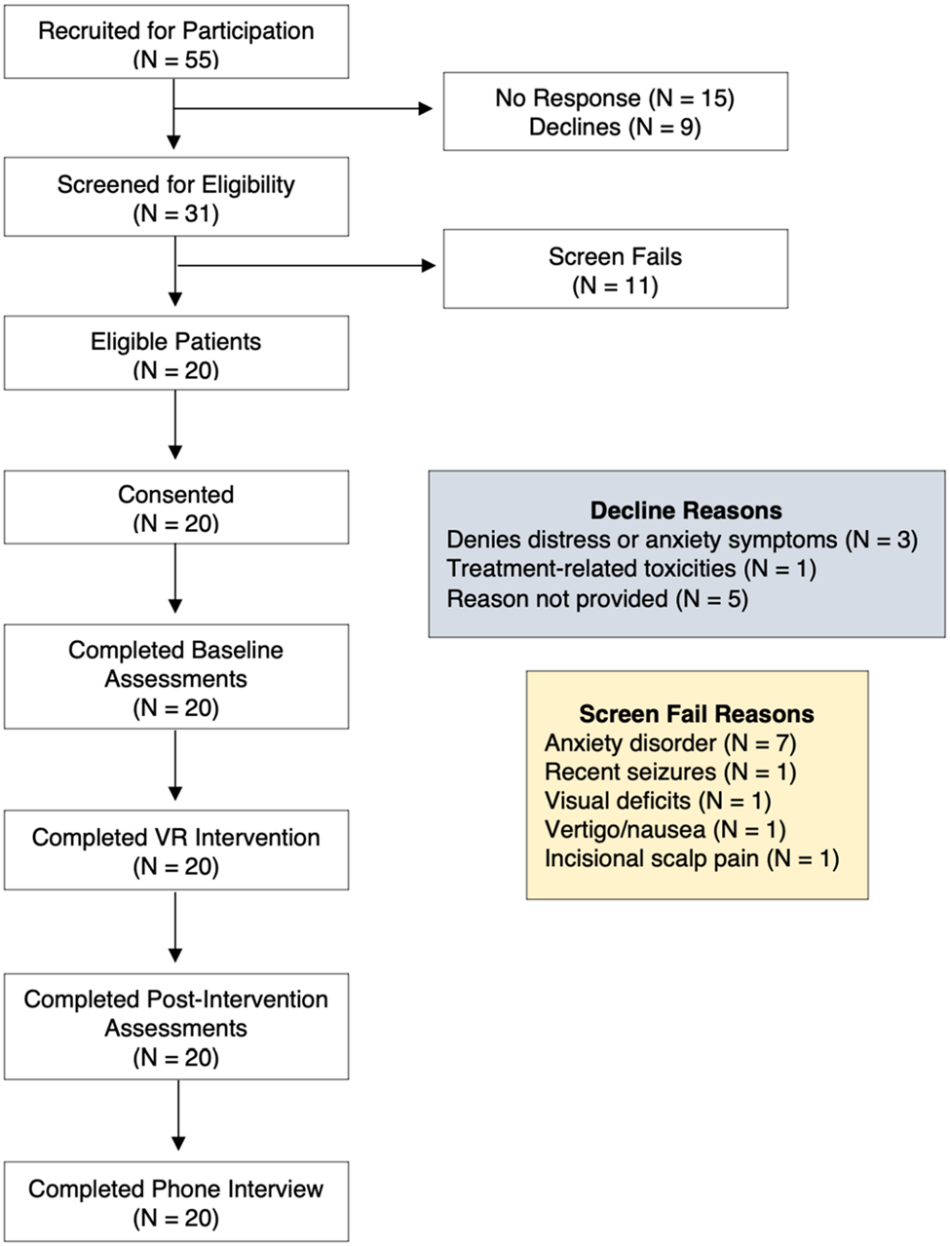
Consort diagram. A total of 55 PBT patients were recruited for participation in this trial (46 via email, 9 in clinic) with 15 patients not responding to the reach-out and 9 patients declined participation (reasons listed above). There were 31 patients who were interested in participating and were screened for eligibility with a total of 11 screen fails (reasons outlined above). Ultimately, 20 patients were eligible and consented to the trial. All enrolled patients completed baseline assessments, the VR intervention, all post-intervention assessments, weekly check-ins with the study team, as well as the qualitative phone interview

Recruitment occurred from March 2021 to March 2022 during the second year of the COVID-19 pandemic. A total of 55 patients who were pre-screened by the research team were approached for participation via email with 15 patients not responding and 9 patients declining participation (Fig. 2). Reasons for patient declines included lack of self-perceived distress or anxiety, treatment-related nausea, or unknown. The remaining 31 patients who were interested in participating were screened for eligibility with 20 patients (65%) deemed eligible and 11 patients (35%) failed screening. The majority of screen fails (7/11) were due to the presence of a pre-existing anxiety disorder, with other exclusionary reasons including recent seizures (1/11), nausea/vertigo (1/11), incisional scalp pain (1/11), and visual deficits (1/11). All 20 patients who were approached and deemed eligible elected to enroll in the trial.

Table 3 shows the patient demographics and clinical characteristics. The mean age of patients was 43 years with 65% of those enrolled < 50 years old. There was an even gender distribution with 100% of patients reporting White race and 15% reporting Hispanic or Latino ethnicity. Most patients had a high-grade tumor (60%) with glioblastoma being the most common diagnosis (30%). Half of the patients were on active treatment and 85% had a good Karnofsky Performance Status (KPS) score at the time of enrollment. The vast majority of patients had low baseline distress (90%), as defined by a score of 0 to 4 on the MD-Anderson Symptom Inventory-Brain Tumor instrument, going into the VR intervention and none were on corticosteroid therapy.

**Table 3:**
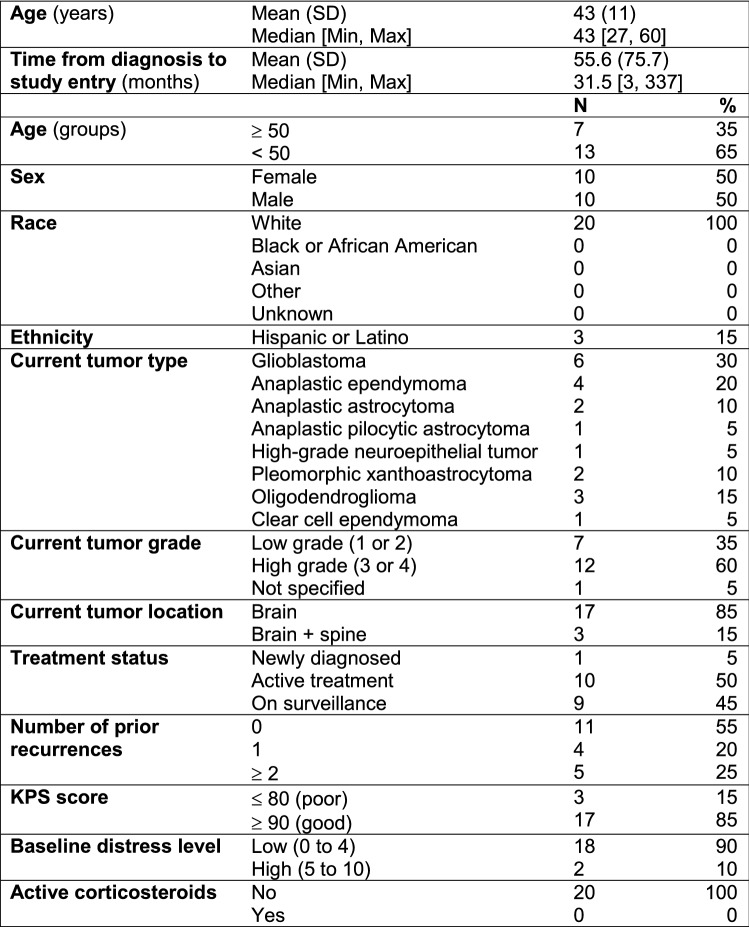
Sample demographics and clinical characteristics (N = 20)

*SD* standard deviation; *KPS* Karnofsky Performance Status

### Feasibility of intervention and procedures

Of the 20 patients who participated in the trial, 100% completed baseline (T_0_) assessments and underwent the initial VR intervention in a telehealth meeting with the study team. Following a demonstration about how to use the headset, all patients were able to navigate use of the device without significant difficulties and completed their first VR scenario under supervision of the research team. 100% of patients completed all post-intervention assessments, including immediately following the first VR intervention (T_1_), 1 week post-intervention (T_2_) and 1 month post-intervention (T_3_) with self-directed ongoing use of the device and completion of the 1 month study period. There were 4 minor deviations due to late completion of questionnaires, which were easily addressed by the study team reaching out electronically to the patient. All patients also took part in the qualitative phone interview 1 week following the initial VR intervention to report their satisfaction.

Out of 20 enrolled patients in the trial, a total of 7 patients (35%) reported mild (grade 1) adverse effects related to use of the VR device, all of which self-resolved following discontinuation of device use during that session. The remaining 13 patients experienced no AEs related to VR device use. The most frequently reported AE was dizziness (3 patients), followed by headache (2 patients), nausea (1 patient), and neck stiffness (1 patient). The 3 patients who reported headache and nausea had just begun chemotherapy treatment. Additionally, the 1 patient who reported neck stiffness was using the VR headset in “gaze mode” while lying down in bed, which likely caused this symptom and resolved when using the remote control for the headset.

### Acceptability of intervention

Acceptability metrics of the VR intervention based on the WIWI questionnaire can be seen in Supplementary Table 1. The majority of patients who participated in this trial reported that it was worthwhile to participate in the VR intervention (90%) and that if they had to do it over again they would use VR in the future (90%). Nineteen out of 20 patients (95%) would recommend VR use to other patients prior to their clinic appointments, which also indicates high acceptability of the intervention. Lastly, 12 of 20 patients (60%) indicated that their quality of life improved following use of VR during their time on study.

## Discussion

Use of VR has the potential to ameliorate some of the negative aspects of cancer that patients endure and allows them to escape to more pleasant environments and experience more positive thoughts and emotions to aid with coping [27]. Within oncology, much of the previous literature has focused on VR interventions that aim to reduce symptoms associated with chemotherapy infusions [28–31], painful procedures such as port or IV placement [32, 33], or targeting distress and other psychological symptoms [27, 34] with promising results to support its use, yet very little information regarding efficacy for patients with brain tumors. This phase 2 feasibility trial is the first known study to assess the feasibility and acceptability of a VR intervention to target distress and anxiety symptoms in this population at the time of clinical evaluation, a time known to cause worsening of psychological symptoms. Results from this interim analysis suggest that this novel interventional strategy is feasible in patients with primary brain tumors and has high reported acceptability to date. We did not attempt to analyze the impact of VR use on distress and anxiety symptoms in this preliminary analysis given the lack of power with such a small sample, though we plan to assess the aforementioned secondary and tertiary study aims in future analyses once accrual and data collection is complete.

Screening and enrollment for this trial proved to be feasible in this population, despite fairly conservative eligibility criteria to ensure safety of enrolled participants. Most patients who were pre-screened and approached for the study were interested in participating. This likely relates to a high prevalence of distress, which was likely heightened during the COVID-19 pandemic [35], and the enthusiasm for an intervention that they could complete at home. The most common reason for screen failures for interested potential patients was having an exclusionary pre-existing anxiety disorder, including generalized anxiety disorder (GAD), post-traumatic stress disorder (PTSD), claustrophobia, and panic disorder. While we did not believe the VR intervention would be harmful to individuals with these conditions, given that this trial aimed to intervene on more situational distress and anxiety at the time of diagnostic imaging, we thought it practical to exclude those with more pervasive anxiety disorders that might be less likely to benefit. Based on the high prevalence of these disorders in our population thus far, we will consider including these individuals in future recruitment in order to assess feasibility and responsiveness to the VR intervention.

Overall, the intervention and study procedures were found to be both feasible and acceptable based on high completion rates for all assessments, zero attrition from the study, and favorable responses to the WIWI questions during the qualitative phone interview. Through weekly check-ins with the patients and study team, we were able to attain 100% data completion for all PROs electronic questionnaires with only 4 deviations for assessments received outside of the pre-determined time windows. Some of the reasons that patients with brain tumors tend to be excluded from clinical trials include a perceived lack of interest in participating in trials or clinician concern for severe cognitive dysfunction in the patients that might prohibit them from following study procedures [36]. We found quite the opposite and despite the majority of patients having high-grade tumors and undergoing active treatment, they were very capable of operating the VR device, following directions for completion of PROs, and were grateful for the opportunity to be a part of this study. Providing patients with a virtual orientation to the device was helpful to address any questions they had upfront and staff were available via email for any questions that arose.

A unique aspect of this trial is that the VR intervention was delivered remotely with patients using the device in their own home. While none of us were used to communicating with patients exclusively via telehealth platforms prior to the COVID-19 pandemic, there were some distinct advantages to this remote approach that may have positively impacted feasibility and acceptability of the VR intervention. Once we all had adapted to using the various virtual platforms, communication with patients was relatively easy there was more flexibility in arranging times for meetings and patients did not have to travel to the NIH to participate. Furthermore, patients were much more likely to be relaxed when using the VR device while in their home environments [37], which may have enhanced the satisfaction with the intervention and potentially augmented any symptomatic improvement they experienced.

A main limitation for this trial is its single-center, non-randomized design. As this is the first VR-based interventional trial in the brain tumor population, it was important to demonstrate feasibility and preliminary efficacy prior to launching a larger randomized study. Additionally, conducting this study during a global pandemic presented challenges for recruitment and also may have biased the types of patients enrolled. Lastly, these findings are from an interim analysis with a relatively small sample and require confirmation in a larger study where feasibility and preliminary efficacy of the intervention can be established. These analyses are planned and will be reported once all patients have been recruited.

In conclusion, findings from this phase 2 trial interim analysis suggest that use of VR to target distress and anxiety symptoms in patients with primary brain tumors at the time of clinical evaluation is both feasible and acceptable and can be administered remotely. Continuation of this trial to collect data from the remaining sample is warranted and will allow further assessment of these feasibility metrics, as well as establishing preliminary efficacy for improving these psychological symptoms in the neuro-oncology population.

## Supplementary Information

Below is the link to the electronic supplementary material.

Supplementary file1 (DOCX 279 KB)

Supplementary file2 (DOCX 191 KB)

Supplementary file3 (DOCX 14 KB)

## Data Availability

The study protocol for this research is posted on ClinicalTrials.gov and additional information or data related to this trial is available from the corresponding author on reasonable request.

## References

[1] National Comprehensive Cancer Network (NCCN) (2002) Distress management. Practice Guidelines in Oncology. Fort Washington, PA: NCCN

[2] Waller A, Groff SL, Hagen N, Bultz BD, Carlson LE (2012). Characterizing distress, the 6th vital sign, in an oncology pain clinic. Palliat Oncol.

[3] Howell D, Olsen K (2008). Distress: the 6th vital sign. Curr Oncol.

[4] Randazzo D, Peters K (2018). Psychosocial distress and its effects on the health-related quality of life for primary brain tumor patients. CNS Oncol.

[5] Liu F, Huang J, Zhang L (2018). Screening for distress in patients with primary brain tumor using distress thermometer: a systematic review and meta-analysis. BMC Cancer.

[6] Otto-Meyer S, Lumibao J, Kim E (2019). The interplay among psychological distress, the immune system, and brain tumor patient outcomes. Curr Opin Behav Sci.

[7] Schuyler D (2004). Cognitive therapy for adjustment disorder in cancer patients. Psychiatry.

[8] Keir S, Calhoun-Eagan R, Swartz J, Saleh O, Friedman H (2007). Screening for distress in patients wtih brain cancer using the NCCN’s rapid screening measure. Psychooncology.

[9] Randazzo D, McSherry F (2017). A cross sectional analysis from a single institution’s experience of psychosocial distress and health-related quality of life in the primary brain tumor population. J Neuro-Oncol.

[10] Lin L, Chiang HH, Acquaye A, Vera-Bolanos E, Gilbert MR, Armstrong TS (2013). Uncertainty, mood states, and symptom distress in patients with primary brain tumors: analysis of a conceptual model using structural equation modeling. Cancer.

[11] Armstrong TS, Vera-Bolanos E, Acquaye A, Gilbert MR, Ladha H, Mendoza T (2016). The symptom burden of primary brain tumors: evidence for a core set of tumor- and treatment-related symptoms. Neuro Oncol.

[12] Bauml JM, Troxel A, Epperson CN (2016). Scan-associated distress in lung cancer: quantifying the impact of “scanxiety”. Lung Cancer.

[13] Cahill J, LoBiondo-Wood G, Bergstrom N, Armstrong TS (2012). Brain tumor symptoms as antecedents to uncertainty: an integrative review. J Nurs Scholarsh.

[14] Maples-Keller JL, Bunnell BE, Kim SJ, Rothbaum BO (2017). The use of virtual reality technology in the treatment of anxiety and other psychiatric disorders. Harv Rev Psychiatry.

[15] Rizzo AA, Buckwalter JG, Neumann U (1997). Virtual reality and cognitive rehabilitation: a brief review of the future. J Head Trauma Rehabil.

[16] Park MJ, Kim DJ, Lee U, Na EJ, Jeon HJ (2019). A literature overview of virtual reality (VR) in treatment of psychiatric disorders: recent advances and limitations. Front Psych.

[17] Leggiero N, Armstrong T, Gilbert M, King A (2020). Use of virtual reality for symptom management in solid-tumor patients with implications for primary brain tumor research: a systematic review. Neuro-Oncol Pract.

[18] National Cancer Institute. Natural history of patients with brain and spinal cord tumors. National Institutes of Health Clinical Center, National Cancer Institute. (https://clinicaltrials.gov/ct2/show/NCT00009035).

[19] Smith A, Mitchell S, Deguiar C (2015). Person-centered outcomes measurement: NIH-supported measurement systems to evaluate self-assessed health, functional performance, and symptomatic toxicity. Transl Behav Med.

[20] Cutillo A, O’Hea E, Person S, Lessard D, Harralson T, Boudreaux E (2017). NCCN distress thermometer: cutt off points and clinical utility. Oncol Nurs Forum.

[21] Marteau TM, Bekker H (1992). The development of a six-item short-form of the state scale of the Spielberger State-Trait Anxiety Inventory (STAI). Br J Clin Psychol.

[22] Tluczek A, Henriques JB, Brown RL (2009). Support for the reliability and validity of a six-item State Anxiety Scale derived from the State-Trait Anxiety Inventory. J Nurs Meas.

[23] Jaremka L, Andridge R, Fagundes C, Alfano C, Povoski S (2014). Pain, depression, and fatigue: loneliness as a longitudinal risk factor. Health Psychol.

[24] Mosher C, Lepore S, Wu L, Valdimarsdottir H (2012). Social correlates of distress following hematopoietic stem cell transplantation: exploring the role of loneliness and cognitive processing. J Health Psychol.

[25] de Souza J, Yap B, Hlubocky F, Wroblewski K, Ratain M (2019). The development of a financial toxicity patient-reported outcome in cancer. Cancer.

[26] Lorenz L, Bachem RC, Maercker A (2016). The adjustment disorder-new module 20 as a screening instrument: cluster analysis and cut-off values. Int J Occup Environ Med.

[27] Mosadeghi S, Reid MW, Martinez B, Rosen BT, Spiegel BM (2016). Feasibility of an immersive virtual reality intervention for hospitalized patients: an observational cohort study. JMIR Mental Health.

[28] Schneider SM, Ellis M, Coombs WT, Shonkwiler EL, Folsom LC (2003). Virtual reality intervention for older women with breast cancer. Cyberpsychol Behav.

[29] Schneider SM, Hood LE (2007). Virtual reality intervention for chemotherapy symptoms. Oncol Nurs Forum.

[30] Schneider SM, Kisby CK, Flint EP (2011). Effect of virtual reality on time perception in patients receiving chemotherapy. Support Care Cancer.

[31] Schneider SM, Prince-Paul M, Allen MJ, Silverman P, Talaba D (2004). Virtual reality as a distraction intervention for women receiving chemotherapy. Oncol Nurs Forum.

[32] Gershon J, Zimand E, Pickering M, Rothbaum BO, Hodges L (2004). A pilot and feasibility study of virtual reality as a distraction for children with cancer. J Am Acad Child Adolesc Psychiatry.

[33] Windich-Biermeier A, Sjoberg I, Dale JC, Eshelman D, Guzzetta CE (2007). Effects of distraction on pain, fear, and distress during venous port access and venipuncture in children and adolescents with cancer. J Pediatr Oncol Nurs.

[34] Espinoza M, Banos RM, Garcia-Palacios A, Wiederhold BK, Riva G (2012). Promotion of emotional wellbeing in oncology inpatients using VR. Annual review of cybertherapy and telemedicine.

[35] Miaskowski C, Paul S, Snowberg K (2020). Stress and symptom burden in oncology patients during the COVID-19 pandemic. J Pain Symptom Manage.

[36] Lee E, Chukwueke U, Hervey-Jumper S (2019). Barriers to accrual and enrollment in brain tumor trials. Neuro Oncol.

[37] Williams R, Bolman C, Lechner L (2019). Online interventions aimed at reducing psychological distress in cancer patients: evidence update and suggestions for future directions. Support Palliative Care.

