# Supplementary material for: Feasibility of a virtual reality intervention targeting distress and anxiety symptoms in patients with primary brain tumors: Interim analysis of a phase 2 clinical trial
Source: J Neurooncol. 2023 Mar 8;162(1):137–45. doi: 10.1007/s11060-023-04271-0 (PMC9993385; doi:10.1007/s11060-023-04271-0)
Supplement: Supplementary file 1 — Supplementary file1 (DOCX 279 KB) [file 11060_2023_4271_MOESM1_ESM.docx]

**
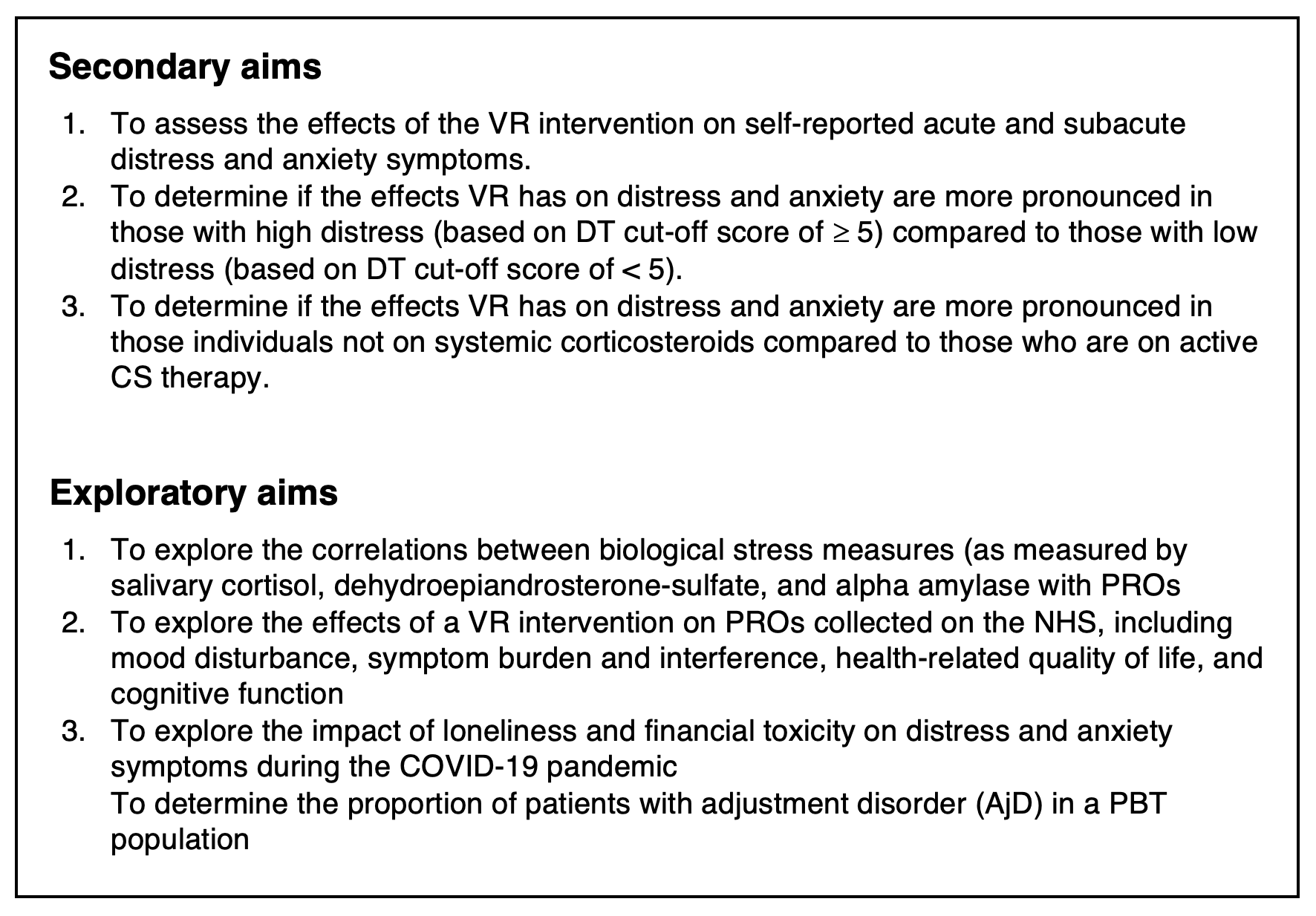
**

**Supplementary Figure 1. Secondary and exploratory aims of the VR trial.** Secondary aims of this trial include assessing the effects of the VR intervention on acute (immediately post-intervention) and subacute (1 to 4 weeks post-intervention) distress and anxiety symptoms while determining if baseline distress levels and corticosteroid use have an impact on intervention efficacy. Exploratory aims assess correlations between salivary stress biomarkers and PROs, VR intervention effects on other PROs assess on the Natural History Study trial, and exploring loneliness, financial toxicity, and prevalence of adjustment disorder.
