# Supplementary material for: Feasibility of a virtual reality intervention targeting distress and anxiety symptoms in patients with primary brain tumors: Interim analysis of a phase 2 clinical trial
Source: J Neurooncol. 2023 Mar 8;162(1):137–45. doi: 10.1007/s11060-023-04271-0 (PMC9993385; doi:10.1007/s11060-023-04271-0)
Supplement: Supplementary file 2 — Supplementary file2 (DOCX 191 KB) [file 11060_2023_4271_MOESM2_ESM.docx]

**
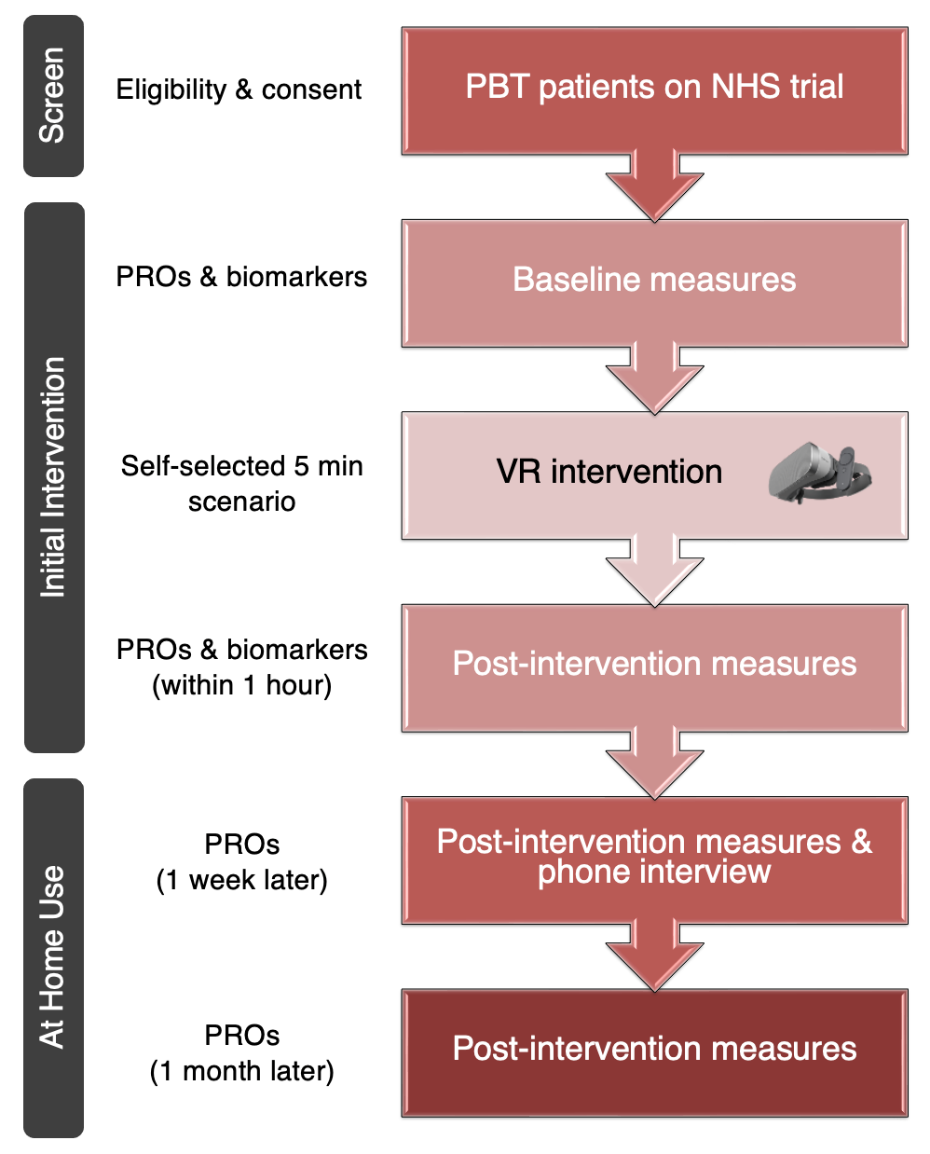
**

Supplementary Figure 2. Phase 2 trial protocol. Adult PBT patients were recruited from the NOB NHS trial at the NIH. Eligible patients were recruited via email or in clinic and completed baseline PROs and optional salivary stress biomarkers within 2 weeks of their clinical evaluation. Research staff met with patients via telehealth to complete an initial VR intervention where participants self-selected a scenario to complete, followed by post-VR intervention assessments within 1 hour to assess acute effects. Patients then continued VR use at home for the 1 month while on study and repeated post-VR intervention assessments at 1 week and 4 weeks to assess subacute effects. A qualitative phone interview was conducted 1 week following the initial VR intervention to assess patient satisfaction.
