# Supplementary material for: Feasibility of a virtual reality intervention targeting distress and anxiety symptoms in patients with primary brain tumors: Interim analysis of a phase 2 clinical trial
Source: J Neurooncol. 2023 Mar 8;162(1):137–45. doi: 10.1007/s11060-023-04271-0 (PMC9993385; doi:10.1007/s11060-023-04271-0)
Supplement: Supplementary file 3 — Supplementary file3 (DOCX 14 KB) [file 11060_2023_4271_MOESM3_ESM.docx]

**Supplementary Table 1. Responses from Was It Worth It (WIWI) questionnaire**

| **Question** | **N** | **Yes (N, %)** | **No (N, %)** |
| --- | --- | --- | --- |
| Was it worthwhile for you to participate in the VR intervention? | 20 | 18, 90% | 2, 10% |
| If you had to do it over, would you use VR again? | 20 | 18, 90% | 2, 10% |
| Would you recommend VR to other patients to use before their clinic appointments? | 20 | 19, 95% | 1, 5% |
| Overall, did your quality of life change by using VR? | 20 | 12, 60% | 8, 40% |
